# Foraging postures are a potential communicative signal in female bonobos

**DOI:** 10.1038/s41598-020-72451-3

**Published:** 2020-09-24

**Authors:** Elisa Demuru, François Pellegrino, Dan Dediu, Florence Levréro

**Affiliations:** 1 Laboratoire Dynamique du Langage, Université de Lyon, CNRS-UMR5596, Lyon, France; 2 Equipe de Neuro-Ethologie Sensorielle ENES/CRNL, Université de Lyon/Saint-Etienne, CNRS-UMR5292, INSERM UMR_S1028, Saint-Etienne, France

**Keywords:** Animal behaviour, Social evolution

## Abstract

Body postures are essential in animal behavioural repertoires and their communicative role has been assessed in a wide array of taxa and contexts. Some body postures function as *amplifiers*, a class of signals that increase the detection likelihood of other signals. While foraging on the ground, bonobos (*Pan paniscus*) can adopt different crouching postures exposing more or less of their genital area. To our knowledge, their potential functional role in the sociosexual life of bonobos has not been assessed yet. Here we show, by analysing more than 2,400 foraging events in 21 captive bonobos, that mature females adopt a rear-exposing posture (*forelimb-crouch*) and do so significantly more often when their anogenital region is swollen than during the non-swollen phase. In contrast, mature males almost completely avoid this posture. Moreover, this strong difference results from a diverging ontogeny between males and females since immature males and females adopt the *forelimb-crouch* at similar frequencies. Our findings suggest that the *forelimb-crouch* posture may play a communicative role of amplification by enhancing the visibility of female sexual swellings, a conspicuous signal that is very attractive for both males and females. Given the high social relevance of this sexual signal, our study emphasizes that postural signalling in primates probably deserves more attention, even outside of reproductive contexts.

## Introduction

Body postures are an essential part of behavioural repertoires in animals and since Darwin’s pioneering work on *The Expression of the Emotions in Man and Animals*^1^ researchers have been interested in their communicative role in a wide array of taxa and behavioural contexts. Some body postures have been interpreted as *amplifiers*^2^, a class of signals that increase the detection likelihood of the information conveyed by other pre-existing signals associated with the signaller’s quality^2,3^. Amplifiers operate in conjunction with the signal they amplify, and although they do not transform it or change its quality (size, strength, colour, etc.), they do increase the probability that it will be perceived by potential receivers. Therefore, an amplifier is specifically selected as a conspicuousness-enhancing “extra signal component” resulting in the emergence of composite signals^4,5^. A classic example of this type of signal has been revealed by Taylor and colleagues^2^ in the jumping spider of the species *Plexippus paykulli*, where males raise their bodies and point their abdomens downwards during interactions with rivals and potential mates. This posture exposes the abdominal pattern, a signal that makes the differences in abdominal width more visible. Other amplifiers have been documented in fish, reptiles, insects, and birds, especially in the context of male courtship [see^4^ for a review].

While postures have long been mentioned as important in great ape communication, they have been overlooked in the recent surge of great ape gesture research^6^ and, to our knowledge, no study has ever considered their potential role as amplifier signals. Here, we aim to fill this gap by examining the role of two foraging postures in bonobos (*Pan paniscus*).

Bonobos present a particularly fascinating repertoire of social behaviours and communicative signals^7^. Among these signals, the exaggerated sexual swelling of female bonobos is probably the most conspicuous. As in other primate species, bonobo females’ anogenital region changes in size and turgidity along the menstrual cycle, becoming a highly noticeable signal during the phase of maximum swelling^8^. However, in contrast with other primates, the maximum sexual swelling in bonobos is not a reliable indicator of ovulation; it has an extremely extended duration and is sexually attractive not only for males but also for females^9,10^. More importantly, this signal is not strictly linked to the reproductive function, but plays an additional role of favouring female socio-sexuality. A species-specific behavioural trait promoting female cohesion^11,12^ is genito-genital (GG) rubbing, in which two bonobo females embrace each-other face to face and rub their genitals together by moving them side to side^13^. Considering the social significance of sexual swelling in this species, it is reasonable to hypothesize that any behaviour enhancing the perceptiveness of this signal should be positively selected.

**Figure 1:**
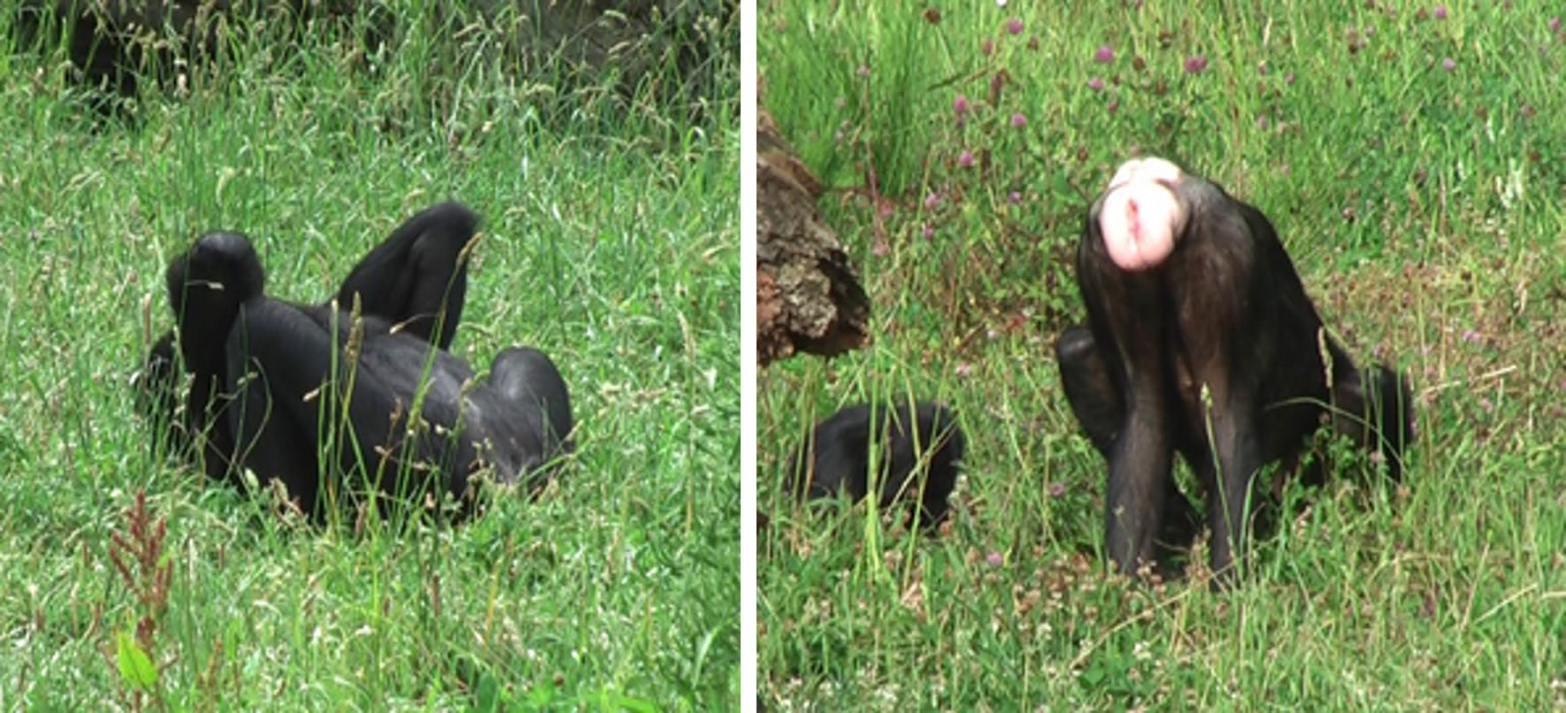
*Full-crouch* (left) and *forelimb-crouch* (right) postures.

While foraging on the ground, bonobos routinely pick up food directly with the mouth adopting two different postures: *full-crouch,* in which elbows and hindlimbs are flexed, and *forelimb-crouch,* in which the elbows are flexed but the knees are not (^14^ and Fig. 1). In contrast with the *full-crouch* posture, the*forelimb-crouch* posture has the peculiarity of exposing the anogenital area, but this postural behaviour has never been investigated as a potential communicative signal. Both postures typically coexist in each individual’s repertoire and, to the best of our knowledge, it is still unknown whether social or ecological factors influence these postural behaviours. In this observational study focussing on the emitter’s perspective, we provide a thorough analysis of the occurrence of *forelimb-crouch* and *full-crouch* in bonobos by putting these two body postures in relation with individual features, such as age, gender, and reproductive and hierarchical status. Knowing the importance of socio-sexual behaviours in bonobo society, our hypothesis is that the foraging posture, by enhancing the visibility of the anogenital swelling, could play a communicative role in the social and reproductive preferences of bonobos and match the definition of *amplifier*^3,4^, which is still sparsely documented in the animal kingdom. We thus predict that the rear-exposing *forelimb-crouch* posture is not equally adopted among individuals. More specifically, we predict that adult females adopt this posture more frequently than adult males and immature subjects, and that this posture is preferred when the sexual swelling is in the maximum phase.

## Materials and methods

### Data acquisition and coding

Data collection was performed at the primate park La Vallée des Singes (France) in June–August 2012, June–July 2014, and April–June 2018, on a total of 21 captive-born bonobos (see supplementary information for group composition). These bonobos lived in a stable social group housed in an enclosure with both an indoor (about 500 m^2^) and outdoor facility (8,500 m^2^). Observations were performed when the animals were in the outside enclosure, consisting of a wooded island surrounded by water canals. Data collection was performed from the moment they left the inside enclosure in the morning (9:00) to the moment they were given access again to the inside enclosure to spend the night (18:30). Before letting the bonobos out, zoo keepers scattered a mixture of seeds and grains on the ground as a form of environmental enrichment. This is the type of food that mostly elicited forelimb- and full-crouching. During the day, the bonobos were fed at 11:30, 14:30, 15:45, 17:00, and 18:00, mainly with roughly chopped vegetables that bonobos eat while sitting or walking. Water was provided ad libitum.

Data collection was observational, complied with the park research charter and had been previously approved by the zoological curator of La Vallée des Singes. Data were collected through continuous video-recordings (~ 380 h) by applying Focus Group sampling^15^, with no specific behaviour being targeted. The analysis encompassed events consisting of bonobos foraging on the ground by picking up small food items (seeds and grains) directly with the mouth. An event began when the bonobo started picking up food from the ground with its mouth by adopting either the *full-crouch* (i.e., elbows and knees are flexed) or the *forelimb-crouch* (i.e., elbows are flexed but the knees are not) posture and lasted until the individual lifted its mouth from the ground for at least 3 s, or if it switched directly from one posture to the other; this rarely occurred (only 7 times out of 2,403 recorded events). For each event, its duration, the individual identity and the body posture (*full-crouch* or *forelimb-crouch*^14^, Fig. 1) were coded by the first author as primary coder. A second researcher, unaware of the aim of the study, independently coded around 25% of randomly chosen events (N = 642) of the total events (N = 2,403). We performed a Cohen’s Kappa analysis that showed almost perfect agreement (98.92% agreement; Cohen’s kappa: 0.98). Aggressive conflicts were recorded by the All Occurrences sampling^15^ method. For each year, and for the mature subjects only, hierarchy was assessed by entering conflicts into a winner/loser socio-matrix, and the rank was estimated by Normalized David's Scores (NDS)^16^. For each year we created hierarchy classes by listing the individual NDS in decreasing order and by categorizing an individual as high ranking (their NDS is in the upper quartile), low ranking (NDS in the lower quartile), and medium ranking (all the others) (see supplementary information for group composition). For each adult female, changes in size, firmness and coloration of the sexual swelling were assessed by the keepers as part of their daily routine since the establishment of the La Vallée des Singes bonobo group in 2009. The bonobo keepers code changes in sexual swelling firmness and size following Furuichi’s method^17^ and distinguish three phases: minimum, intermediate and maximum. The intermediate phase encompasses both increasing (i.e., from minimum to maximum) and decreasing swelling size (i.e., from maximum to minimum) and, therefore, it does not represent a homogenous category. We decided to keep the intermediate phase in the analyses to provide a general overview of the phenomenon. The keepers were unaware of the aim of the study.

### Statistics

We used mixed-effects logistic regression as implemented by R’s^18^glmer() function in the lme4 package, where the *posture* in each foraging event, coded as “down” (= *full-crouch*) or “up” (= *forelimb-crouch*), is the *dependent variable*, and the *subject* and *year_collection* are *random effects*. The interpretation of the fixed effect slopes*β* is in terms of increasing (*β* > 0) or decreasing (*β* < 0) the probability of an “up” posture. For the first model (all subjects), the fixed effects considered were *sex* (female vs male), *age* (in years), *age_class* (immature vs mature), and *duration* (the natural logarithm of the feeding episode’s duration in seconds). For the second model (mature females only), we considered as fixed effects *age*, *swelling* (a three-level ordered factor “min” < “intermediate” < “max”), *hierarchy* (ordered factor: “high” > “intermediate” > “low”) and *duration* (as above). For both models, we first tested each potential predictor separately, and we only retained those resulting in a significant improvement (at the liberal *α*-level of 0.10) in predicting *posture* over the null model (including only the intercept and the random effects); importantly, *hierarchy* was never retained, as it did not make a significant contribution on its own; also, *age* and *age_class* were never simultaneously considered in the same model. We then used model simplification of the full model including all these retained predictors (and their interactions), iteratively removing the interactions and predictors that failed to make a significant (at *α*-level 0.05) contribution (for full details please see the accompanying Rmarkdown). With these, the final models for all individuals, and of mature females only, are, respectively (in R formula notation):

$${\text{posture }}\sim {\text{ sex }} + {\text{ duration }} + {\text{ age}}\_{\text{class }} + {\text{ sex }}:{\text{ duration }} + {\text{ sex }}:{\text{ age}}\_{\text{class }} + \, \left( {{1 }|{\text{ subject}}} \right) \, + \, \left( {{1 }|{\text{ year}}\_{\text{collection}}} \right)$$

and:

$${\text{posture }}\sim {\text{ swelling }} + {\text{ duration }} + \, \left( {{1 }|{\text{ subject}}} \right) \, + \, \left( {{1 }|{\text{ year}}\_{\text{collection}}} \right)$$

## Results

We sampled 2,403 foraging events from video recordings of 21 bonobos and statistically modelled them, with posture (*forelimb-crouch* or *full-crouch*) as the dependent variable (see “Materials and methods” and Table 1. See also the dataset, descriptive data, and detailed statistical analyses provided in the supplementary information).

**Table 1:** Summary of statistical models, showing fit indices (marginal R^2^ and Akaike Information Criterion AIC), the between-group variances for the random effects (τ_00_ for subject and year_collection), and point estimates, 95% confidence intervals and p-values of predictors and their interactions (if included).

Model	Predictor	Estimate	95% CI	p	p*
All individuals R^2^ = 40.2% AIC = 2,325.9 *τ*_00 subject_ = 0.61 *τ*_00 year_collection_ = 0.16	Intercept	0.13	(− 0.77, 1.02)	0.78	–
	Sex (M)	0.63	(− 0.51, 1.76)	0.28	–
	Duration	− **0.56**	**(**− **0.68, **− **0.43)**	** < 2.16 × 10**^**–16**^	–
	Age_class (mature)	**1.03**	**(0.08, 1.98)**	**0.034**	–
	Sex (M) : duration	− **0.72**	**(**− **1.01, **− **0.42)**	**1.99 × 10**^**–6**^	**5.21 × 10**^**–7**^
	Sex (M) : age_class (mature)	− **2.31**	**(**− **3.60, **− **1.02)**	**0.0004**	**0.0016**
Mature females R^2^ = 20.2% AIC = 942.9 *τ*_00 subject_ = 0.20 *τ*_00 year_collection_ = 0.41	Intercept	**1.51**	**(0.59, 2.43)**	**0.0013**	–
	Swelling (linear)	**1.26**	**(0.89, 1.63)**	**2.59 × 10**^**–11**^	**2.73 × 10**^**–13**^
	Swelling (quadratic)	− **0.47**	**(**− **0.86, **− **0.08)**	**0.018**	
	Duration	− **0.55**	**(**− **0.72, **− **0.39)**	**9.69 × 10**^**–11**^	–

The estimates and 95% CIs are in log odds of the position being “up”. For interactions in the “all individuals” model, each p* is the p-value of the whole interaction, while for the “mature females” model, p* is the p-value of the whole linear and quadratic effects. Bold = significance at α-level 0.05.

**Figure 2:**
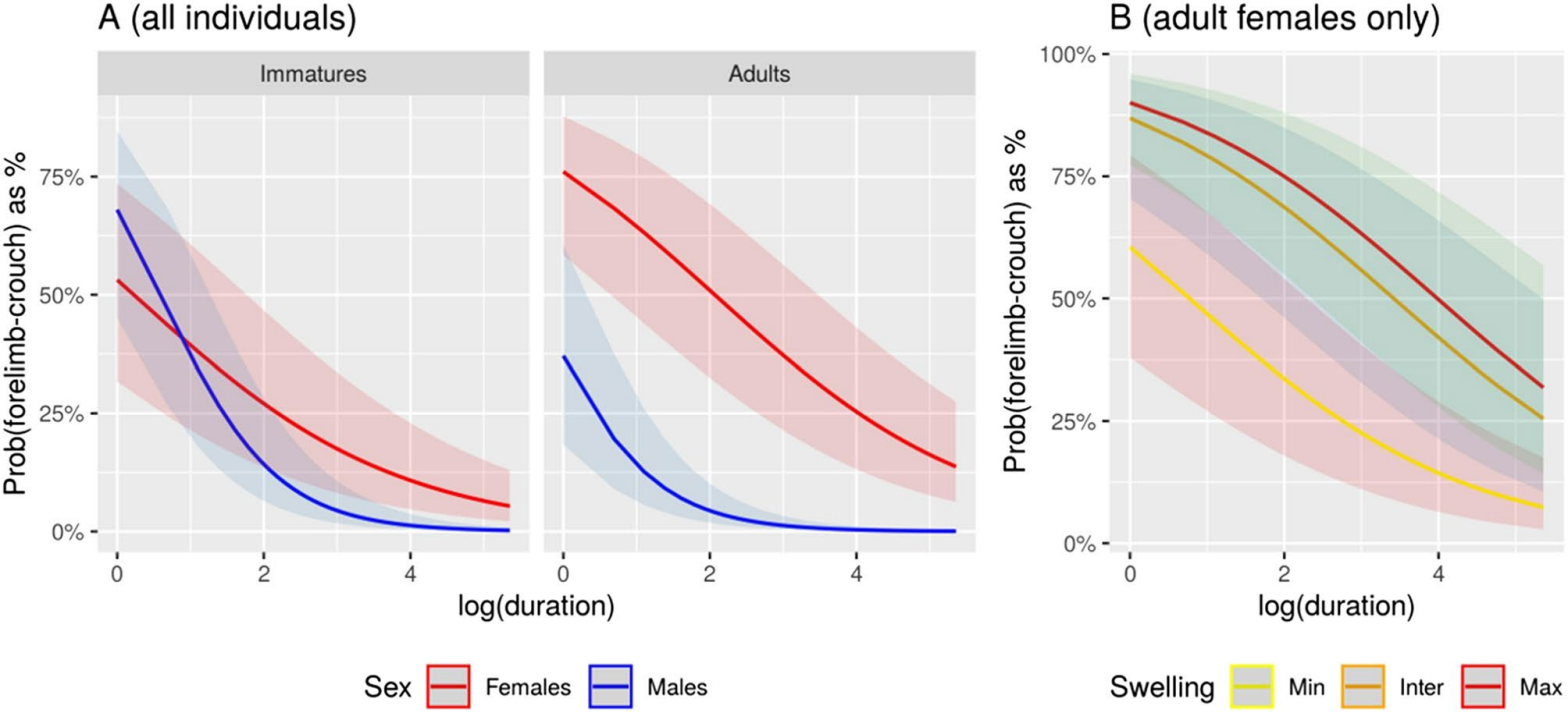
Panels displaying the percent probability of forelimb-crouching and the log(duration) for three subgroups of subjects: **(a)** by sex for immatures and adults, and **(b)** by swelling phase for mature females.

The statistical analysis shows a diverging ontogeny between males and females in their probability of forelimb-crouching (Fig. 2a): forelimb-crouching seldom occurs in mature males compared to immature males (6.3% vs 30.2% of observed events respectively), whereas this posture is more often displayed in mature (58.9%) than immature (40.3%) females. There is indeed a significant positive effect of being *mature* on the probability of forelimb-crouching (*β* = 1.03,*p* = 0.034) and a significant interaction between*sex* and *age class* (*p* = 0.0016). Event duration is also influential, with most individuals showing a preference for full-crouching for longer durations (*β* = –0.56, *p* < 2.16∙10^–16^), but with a higher probability of *forelimb-crouch* posture for females (*p* = 1.99∙10^–6^).

Focusing on mature females (Fig. 2b), we found that while their posture is not affected by hierarchical rank (*p* = 0.71), it does change with swelling (*p* = 2.73∙10^–13^): at intermediate and maximum swelling phases there is a higher probability of forelimb-crouching (69.0% and 66.3%) than at minimum swelling phase (49.6%), this relationship being non-linear (linear component: *β* = 1.26, *p* = 2.59∙10^–11^, and quadratic component:*β* = –0.47, *p* = 0.018; Table 1). The negative effect of *duration* is confirmed in this second analysis (*β* = –0.55, *p* = 9.69∙10^–11^).

## Discussion

Our results show that whereas immature bonobos did not show any significant sex differences in positional preference, mature bonobos displayed strong preferences either to adopting the rear-exposing *forelimb-crouch* (females) or to avoiding it (males). For mature females, the changes in their postural behaviours through their sexual swelling cycle suggest a stronger preference for forelimb-crouching during the swelling phases (i.e.*,* intermediate and maximum) over the non-swelling phase. In all cases, individuals engaging in foraging events longer than 12 s tended to adopt the*full-crouch* posture, which is compatible with the physiological cost of ingesting food with an upside-down digestive tract. These findings outline a general pattern with a random distribution between forelimb-crouching and full-crouching for immature subjects and for mature females in the minimal swelling phase. In contrast, the dichotomy observed between mature females in swollen phases and mature males strongly suggests a non-random sex-dependent cost–benefit ratio. The infrequency of *forelimb-crouch* in mature males may be driven by the need to reduce the risk of exposing the testicles, a body part often targeted during conflicts in the *Pan* species^19,20^. For mature females, the fact that dominance rank did not influence the probability of *forelimb-crouch* lets us reject the competing explanation that this posture might represent a hierarchy signal^21^. Moreover, the fact that a general preference for full-crouching for longer foraging events was also observed in females during the maximum swelling phase rules out the competing explanation that this posture is adopted because the swelling hinders full-crouching.

Taken together, our results support the hypothesis that the *forelimb-crouch* posture represents a low-cost means of improving the signalling efficiency of sexual swelling in bonobos. As mentioned above, bonobo maximum swelling has an extremely prolonged duration and is not a reliable indicator of ovulation, resulting in a decreased reliability of bonobo maximum swelling as a fecundity signal, which, in turn, influences male mating strategies by increasing the costs of mate-guarding to ascertain paternity^9^. It is acknowledged that the features of female swelling contribute to shaping bonobo peaceful society by decreasing the level of male competition^22^. Moreover, this swelling signal is also sexually attractive for females, and it has been proposed that its prolonged duration was selected to increase female cohesiveness^11^. Bonobo females are indeed unique among primates for the extensive use of same-sex sexual behaviour. Sexual interactions in female dyads (but not in mixed-sex dyads) increase their urinary level of oxytocin, a hormone promoting social bonding and cooperation^12^ and allow them to limit male harassment and to reach high hierarchical positions in a male philopatric society^23^. Therefore, broadcasting their sexual swelling signal to the highest number of potential receivers, both males and females, appears essential for bonobo females. In this sense, the *forelimb-crouch* posture may be interpreted as an *amplifier* signal of sexual swelling, the combination of posture and sexual swelling resulting in a unimodal (visual) composite signal^24^. Although several physical or behavioural amplifiers have been shown to enhance visual salience, and the *Amplifier Hypothesis* proposed by Oren Hasson in 1989^3^ has been advocated in theoretical and computational models^25–27^, empirical studies are still rare, limited to a few taxa and to the context of courtship and reproduction^28–30^. Therefore, the *Amplifier Hypothesis* is still debated^27^, but models predict that any cost-limited amplifier that enhances the perception of a relevant signal should be under positive selection. This strongly suggests that the existence of amplifiers may have been overlooked in natural behaviours and that we also need to provide empirical case studies covering a wider array of behavioural contexts and species, in parallel with clearer theoretical and terminological positions.

The diverging ontogeny of forelimb-crouching between males and females revealed in our study supports the hypothesis that the *forelimb-crouch* posture has been selected in female emitters for its functional role in the regulation of bonobo society. In particular, the decrease in the frequency of *forelimb-crouch* in adult males is highly compatible with the need of protecting their genitals from possible attacks^19,20^. In contrast, adult females increase their tendency to adopt this posture and our data support the view that they do so to increase the visibility of their swollen anogenital region. The interpretation of the *forelimb-crouch* posture as a visual amplifier of sexual swelling in bonobos would broaden the current empirical evidence on amplifiers, by providing three novel characteristics: it amplifies a *female* sexual signal *outside* the reproductive context by increasing its conspicuousness for a *large audience*. Moreover, this would be the first time a postural amplifier is discovered in primates.

By focussing on postural signal production, our study addresses an aspect that has been overlooked so far in primates. Our results encourage further studies evaluating potential audience effects, as well as male and female receivers’ attention and behavioural responses to confirm that this posture has a communicative function aimed at enhancing swelling visibility in bonobos. There is as yet no evidence that the*forelimb-crouch* is used as a sexual invitation and we do not believe that this posture is an overt sexual solicitation. It is primarily a foraging posture that highlights the genital parts and thus the sexual swelling stage of adult females. Because of the lack of data in situ, it is currently difficult to assess the efficiency of such visual amplification. Although bonobos live in forest habitats, these are of heterogeneous types. Whereas previous studies on wild bonobos have mainly been conducted in rich tropical rainforests, it has been recently confirmed that wild bonobos inhabit a wider range of environments than previously thought, and notably forest-savanna mosaic environments^31–34^, and the efficient range in the visual channel thus varies among these forest habitat types. In line with the importance of visual communication in bonobos (e.g. gestures^35^), a potential amplifier role of forelimb-crouching is thus possible and our unambiguous results in captivity are consistent with this hypothesis. However, we cannot exclude that captivity has favoured this behaviour since (i) the visibility between individuals is almost permanent and (ii) bonobos spend more time on the ground foraging than in the wild (F. Levréro’s personal communication). In addition, we suggest that the *forelimb-crouch* posture might be present in other primate species showing sexual swelling, where it might enhance this more reliable signal of fertility. More generally, by focussing on human’s closest living relatives our study bridges theoretical and empirical aspects and sheds light on postural signalling as a potential neglected dimension of the evolutionary pathways leading to improved communication efficiency in animals, including humans.

## Supplementary information

Supplementary Information 1.

Supplementary information 2.

Supplementary information 3.

## Data Availability

Dataset is provided in the supplementary information as well as the Rmarkdown script and html output of the statistical analyses.
