# Supplementary material for: Foraging postures are a potential communicative signal in female bonobos
Source: Sci Rep. 2020 Sep 24;10:15431. doi: 10.1038/s41598-020-72451-3 (PMC7608273; doi:10.1038/s41598-020-72451-3)
Supplement: Supplementary file 2 — Supplementary information 2. [file 41598_2020_72451_MOESM2_ESM.html]

Foraging postures are a potential communicative signal in female Bonobos


# Foraging postures are a potential communicative signal in female Bonobos

### – the script for generating the plots, summaries and statistical analyses –

#### Elisa Demuru, François Pellegrino, Dan Dediu [ddediu@gmail.com] & Florence Levréro

#### 05 July, 2020

## Introduction

This `Rmarkdown` script contains all the code necessary for reproducing the plots and statistical analyses reported in the *Foraging postures are a potential communicative signal in female Bonobos* paper; it also contains a few more analyses that could not be included due to space constraints, as well as more details about the analyses and the methodological decisions.

## The data

The data is contained in the `Dataset_Demuru_et_al.csv` TAB-separated CSV file, and is structured as follows (please note that we have renamed the variables, so they have shorter names for these analyses; also, some variables are not used at all):

| Original name | Short name | Description/details | Used for analyses? |
| --- | --- | --- | --- |
| DATE DATA COLLECTION | *date\_collection* | the actual date (in mm/dd/yyyy format) when the observation was made | yes |
| DATE BIRTH | *date\_birth* | the individual’s date of birth (in mm/dd/yyyy format) | no (use *age* instead) |
| SUBJECT ID | *subject* | the individual’s unique id (anonymized); please note that we use “subject” and “individual” interchangeably in the text | yes |
| POSTURE (0 = down, 1 = up) | *posture* | the feeding posture (recoded as a factor with levels *up* and *down*; **please note that**, for brevity, *up* is a shorthand for *forelimb crouch* and *down* for *full crouch*) | yes |
| DURATION (hh:mm:ss) | *duration.hms* | the duration of the observation in the hours:minutes:sconds format | no (use *duration* instead) |
| DURATION (in seconds) | *duration* | the duration of the observation in sconds (**please note that** while for the plots the duration is as such, for the models we use `log(duration)`) | yes |
| YEAR DATA COLLECTION | *year\_collection* | the year of the data collection (**please note that** each year may be different in several respects) | yes |
| YEAR BIRTH | *year\_birth* | the year the individual was born | no (using *age* instead) |
| AGE | *age* | the subject’s age when the observation was made (in years) | yes |
| AGE CLASS (0 = immature, 1 = mature) | *age\_class* | is the subject *immature* or *mature* (factor with two levels) | yes |
| SWELLING (1 = minimum, 2 = intermediate, 3 = maximum) | *swelling* | the level of swelling as an ordered factor with levels 1, 2 and 3 | yes |
| SEX (F= female, M = male) | *sex* | the individual’s sex (factor with levels *F* and *M*) | yes |
| HIERARCHY (NDS scores) | *hierarchy* | the actual NDS numbers (comparable within a year but not across years) | yes |
| HIERARCHY (1 = low, 2 = intermediate, 3 = high) | *hierarchy\_class* | the hierarchy category (as an ordered factor with levels *low*, *intermediate*, *high*); can be compared between years | yes |

## Data summaries

### Overall

|  | all\_data (N = 2,403) |
| --- | --- |
| **Posture** |  |
| up | 875 (36%) |
| down | 1,528 (64%) |
| **Duration** |  |
| min | 1 |
| median (iqr) | 8 (4.00, 16.00) |
| mean (sd) | 13.95 ± 19.23 |
| max | 212 |
| **Year collection** |  |
| 2012 | 703 (29%) |
| 2014 | 403 (17%) |
| 2018 | 1,297 (54%) |
| **Age** |  |
| min | 1 |
| median (iqr) | 9 (6.00, 17.00) |
| mean (sd) | 12.00 ± 8.83 |
| max | 50 |
| **Age class** |  |
| immature | 997 (41%) |
| mature | 1,406 (59%) |
| **Sex** |  |
| female | 1,423 (59%) |
| male | 980 (41%) |
| **Swelling (mature females)** |  |
| 1 | 381 (47%) |
| 2 | 158 (20%) |
| 3 | 264 (33%) |
| **Hierarchy (mature individuals)** |  |
| min | 1.479 |
| median (iqr) | 1,406; 5.11 (3.60, 5.89) |
| mean (sd) | 1,406; 4.58 ± 1.37 |
| max | 6.948 |
| **Hierarchy class (mature individuals)** |  |
| low | 272 (19%) |
| intermediate | 770 (55%) |
| high | 364 (26%) |

### By sex

|  | sex: F (N = 1,423) | sex: M (N = 980) |
| --- | --- | --- |
| **Posture** |  |  |
| up | 723 (51%) | 152 (16%) |
| down | 700 (49%) | 828 (84%) |
| **Duration** |  |  |
| min | 1 | 1 |
| median (iqr) | 7 (4.00, 14.00) | 9.00 (4.00, 18.00) |
| mean (sd) | 12.24 ± 15.51 | 16.42 ± 23.39 |
| max | 212 | 208 |
| **Year collection** |  |  |
| 2012 | 405 (28%) | 298 (30%) |
| 2014 | 174 (12%) | 229 (23%) |
| 2018 | 844 (59%) | 453 (46%) |
| **Age** |  |  |
| min | 1 | 2 |
| median (iqr) | 9 (5.00, 17.00) | 11.00 (6.00, 20.00) |
| mean (sd) | 11.60 ± 9.96 | 12.58 ± 6.83 |
| max | 50 | 23 |
| **Age class** |  |  |
| immature | 620 (44%) | 377 (38%) |
| mature | 803 (56%) | 603 (62%) |
| **Sex** |  |  |
| female | 1,423 (100%) | 0 (0%) |
| male | 0 (0%) | 980 (100%) |
| **Swelling (mature females)** |  |  |
| 1 | 381 (47%) | 0 (NaN%) |
| 2 | 158 (20%) | 0 (NaN%) |
| 3 | 264 (33%) | 0 (NaN%) |
| **Hierarchy (mature individuals)** |  |  |
| min | 1.479 | 2.427 |
| median (iqr) | 803; 5.13 (3.77, 6.00) | 603; 4.37 (3.60, 5.11) |
| mean (sd) | 803; 4.71 ± 1.55 | 603; 4.39 ± 1.07 |
| max | 6.948 | 6.903 |
| **Hierarchy class (mature individuals)** |  |  |
| low | 110 (14%) | 162 (27%) |
| intermediate | 395 (49%) | 375 (62%) |
| high | 298 (37%) | 66 (11%) |

### By age class

Please note that for *immatures*, the summaries for *swelling*, *hierarchy* and *hierrchy class* produce, by definition, nonsensical answerws (i.e., `0`, `NaN` and `±Inf`).

|  | age\_class: immature (N = 997) | age\_class: mature (N = 1,406) |
| --- | --- | --- |
| **Posture** |  |  |
| up | 364 (37%) | 511 (36%) |
| down | 633 (63%) | 895 (64%) |
| **Duration** |  |  |
| min | 1 | 1 |
| median (iqr) | 7 (4.00, 13.00) | 8.00 (4.00, 18.00) |
| mean (sd) | 11.78 ± 16.45 | 15.48 ± 20.84 |
| max | 170 | 212 |
| **Year collection** |  |  |
| 2012 | 239 (24%) | 464 (33%) |
| 2014 | 189 (19%) | 214 (15%) |
| 2018 | 569 (57%) | 728 (52%) |
| **Age** |  |  |
| min | 1 | 9 |
| median (iqr) | 5 (4.00, 6.00) | 17.00 (11.00, 20.00) |
| mean (sd) | 4.94 ± 1.71 | 17.00 ± 8.41 |
| max | 8 | 50 |
| **Age class** |  |  |
| immature | 997 (100%) | 0 (0%) |
| mature | 0 (0%) | 1,406 (100%) |
| **Sex** |  |  |
| female | 620 (62%) | 803 (57%) |
| male | 377 (38%) | 603 (43%) |
| **Swelling (mature females)** |  |  |
| 1 | 0 (NaN%) | 381 (47%) |
| 2 | 0 (NaN%) | 158 (20%) |
| 3 | 0 (NaN%) | 264 (33%) |
| **Hierarchy (mature individuals)** |  |  |
| min | Inf | 1.479 |
| median (iqr) | 0; NA ( NA, NA) | 5.11 (3.60, 5.89) |
| mean (sd) | 0; NaN ± NA | 4.58 ± 1.37 |
| max | -Inf | 6.948 |
| **Hierarchy class (mature individuals)** |  |  |
| low | 0 (NaN%) | 272 (19%) |
| intermediate | 0 (NaN%) | 770 (55%) |
| high | 0 (NaN%) | 364 (26%) |

### For mature females only

|  | mature\_females (N = 803) |
| --- | --- |
| **Posture** |  |
| up | 473 (59%) |
| down | 330 (41%) |
| **Duration** |  |
| min | 1 |
| median (iqr) | 7 (3.50, 16.50) |
| mean (sd) | 13.61 ± 17.49 |
| max | 212 |
| **Year collection** |  |
| 2012 | 233 (29%) |
| 2014 | 75 (9%) |
| 2018 | 495 (62%) |
| **Age** |  |
| min | 9 |
| median (iqr) | 17 (9.00, 17.00) |
| mean (sd) | 16.92 ± 10.42 |
| max | 50 |
| **Swelling** |  |
| 1 | 381 (47%) |
| 2 | 158 (20%) |
| 3 | 264 (33%) |
| **Hierarchy** |  |
| min | 1.479 |
| median (iqr) | 5.13 (3.77, 6.00) |
| mean (sd) | 4.71 ± 1.55 |
| max | 6.948 |
| **Hierarchy class** |  |
| low | 110 (14%) |
| intermediate | 395 (49%) |
| high | 298 (37%) |

## Exploratory plots

Here we show various exploratory views of the data, per individual and across individuals.

### Per individual

#### Posture and duration by swelling level for adult females

***Figure 1.*** *Duration and posture by swelling level for adult females (split by individual).*

***Figure 2.*** *Posture by swelling level for adult females (split by individual and observation year).*

#### Posture and duration for juveniles and adult males

***Figure 3.*** *Duration and posture for adult males and juveniles (split by individual).*

***Figure 4.*** *Posture for adult males and juveniles (split by individual and observation year).*

### Across individuals

#### Posture, duration and sex

***Figure 5.*** *Posture, duration and sex.*

#### Posture, age and sex

***Figure 6.*** *Posture, age and sex.*

#### Posture, hierarchy and sex

***Figure 7.*** *Posture, hierarchy and sex.*

#### Posture, hierarchy and swelling for mature females only

***Figure 8.*** *Posture, hierarchy and swelling for mature females only.*

#### Posture, hierarchy, age and sex

***Figure 9.*** *Posture, hierarchy, age and sex.*

#### Posture, hierarchy, duration and sex

***Figure 10.*** *Posture, hierarchy, duration and sex.*

#### Posture, hierarchy\_class, age and sex

***Figure 11.*** *Posture, hierarchy\_class, age and sex.*

#### Posture, hierarchy\_class, duration and sex

***Figure 12.*** *Posture, hierarchy\_class, duration and sex.*

## Statistical analyses

The *variables* are coded as follows:

- *sex* is coded as *F* vs *M*;
- *posture* is coded as *down* vs *up*, so that the logistic models represent the probability of an *up* position;
- *age\_class* is coded *immature* vs *mature*;
- *swelling* is considered as ordered factor (1 < 2 < 3);
- *age* is considered as numeric;
- *duration* is considered as numeric, and we took its `log` (to improve its distribution);
- *year\_collection* can be used a proxy for “cohort”;
- *hierarchy* is numeric and quite precise but cannot be compared across cohorts, while *hierarchy\_class* (ordered factor) is much less precise but in principle should be comparable across cohorts.

*The modelling*:

- we use *sum contrasts* for factors, except for the *ordered* factors (*swelling* and *hierarchy\_class*), for which we use *ploynomial contrasts* (see note 1 for details on interpretating contrasts)
- we use throughout *mixed-effects logistic regressions* as implemented by `glmer()`;
- the DV is always *posture* (*down* is 0 and *up* is 1);
- we model both *subject* and *year\_collection* as random effects (to capture both indvidual-specific and year/cohort-specific effects);
- the approach we use is:

  1. fit first the *null* model with only the intercept and the random effects (e.g., `posture ~ 1 + (1 | subject) + (1 | year_collection)`);
  2. determine which of the potential IVs make an individual singificant contribution by comparing the *null* model with the *null* + the single IV at the liberal *α*-level 0.1 (we are liberal here as we would rather not miss potentially important predictors and as this process is anyway followed by a selection procedure at the usual alpha level of 0.05); for example, we compare the *null* model `posture ~ 1 + (1 | subject) + (1 | year_collection)` to the “sex-only” model `posture ~ 1 + sex + (1 | subject) + (1 | year_collection)` and retain *sex* as a potential IV only if the second model is significantly better than the *null* as judged by an `anova()` test at the *α*-level 0.1;
  3. we fit the *full* model contaning all the IVs selected in step (2), their interactions with varying intercept(s) (e.g., if we selected *sex* and *duration* as individually making significant contributions, the *full* model is `posture ~ 1 + sex * duration + (1 | subject) + (1 | year_collection)`);
  4. finally, we perform model simplification, stopping when the fit becomes significantly worse (e.g., for our example, we migt stop at the *final* model `posture ~ 1 duration + (1 | subject) + (1 | year_collection)`).

This “heterogeneous” approach allows us to obtain the simplest models that still explain the data well enough, while avoiding starting with extremely complex models (which may have convergence problems or be extremely slow to converge).

*Time dependency* and *autocorrelation*:

Unfortunately, modelling temporal autocorrelation with `glmer()` is not obvious, but we checked the residuals of the *final* models for autocorrelations (using the `acf()` function), and in most cases there’s no autocorrelation, and even when there is a detectable autocorrelation this is small and decays rapidly.

As a supplementary check (not shown here), we also modelled time dependency by including as an IV the number of days since the begining of the year/cohort, but this is rarely retained as a significant predictor, and, even when it is, the effects of the other predictors and the residual autocorrelations are virtually unchanged, prompting us to ignore this in the final analysis presented here.

*Outliers*:

We identified and removed strong outliers, but the results remain very similar whether the outliers are removed or not.

*Modelling hierarchy*:

We modeled hierarchy as as the numeric NDS scores (variable *hierarchy*) or as the ordered categorical variable *hierarchy\_class*, but the results are identical in the sense that hierarchy is mever retained as a potential explanatory factor in whichever form.

### Is posture clustered by individual and observation year?

ICC of *posture* by *subject* is 29.6% and by *year\_collection* is 5.7%, so we use both *subject* and *year\_collection* as random effects.

### Mature females only

#### Outliers:

We identified and removed 2 outliers; in order of removal, these are:

|  | date\_collection | subject | posture | duration | age | swelling | year\_collection |
| --- | --- | --- | --- | --- | --- | --- | --- |
| 526 | 2012-07-25 | 18 | up | 4.1108739 | 27 | 1 | 2012 |
| 1795 | 2018-05-25 | 7 | down | 0.6931472 | 17 | 2 | 2018 |

#### Summaries:

The distribution of postures across individuals (with column and row sums) is:

|  | down | up | Sum |
| --- | --- | --- | --- |
| **2** | 17 | 44 | 61 |
| **7** | 106 | 239 | 345 |
| **10** | 75 | 64 | 139 |
| **13** | 60 | 50 | 110 |
| **18** | 8 | 5 | 13 |
| **19** | 38 | 22 | 60 |
| **20** | 25 | 48 | 73 |
| **Sum** | 329 | 472 | 801 |

#### Retained *IV*s:

The potential IVs (each tested individually for their contribution to *posture*):

Individual contribution of all potential IVs to *posture*. We only keep the ones with a significant individual contribution (column *kept*) for further modelling.

| IV | beta | p | AIC | kept |
| --- | --- | --- | --- | --- |
| 1 | NA | NA | 1051.6 | yes |
| age | 0.011 | 0.4460 | 1053.0 | no |
| swelling | 1.220 | 0.0000 | 993.1 | yes |
| hierarchy | 0.042 | 0.7117 | 1053.5 | no |
| duration | -0.564 | 0.0000 | 1003.4 | yes |

#### Best model:

The best model (with diagnostic plots) is:

posture ~ swelling + duration + (1 | subject) + (1 | year\_collection)

|  | posture | | |
| --- | --- | --- | --- |
| Predictors | Log-Odds | CI | p |
| (Intercept) | 1.51 | 0.59 – 2.43 | **0.001** |
| swelling.L | 1.26 | 0.89 – 1.63 | **<0.001** |
| swelling.Q | -0.47 | -0.86 – -0.08 | **0.018** |
| duration | -0.55 | -0.72 – -0.39 | **<0.001** |
| Random Effects | | | |
| σ2 | 3.29 | | || τ00 subject | 0.20 | | || τ00 year\_collection | 0.41 | | || ICC | 0.16 | | || N subject | 7 | | || N year\_collection | 3 | | || Observations | 801 | | |
| Marginal R2 / Conditional R2 | 0.202 / 0.328 | | |
| Deviance | 930.958 | | |
| AIC | 942.958 | | |
| log-Likelihood | -465.479 | | |

The effect of *swelling* is highly significant with *p* = \(2.7246419\times 10^{-13}\).

***Figure 13.*** *Model diagnostic plot (NB: this is on the *link scale*).*

***Figure 14.*** *Autocorrelation of the model’s residuals.*

The best model’s predictions are:

***Figure 15.*** *Predicted probability of *up* (y axis) function of *swelling* for mature females.*

***Figure 16.*** *Predicted probability of *up* (y axis) function of *duration* (x axis) and *swelling* (colors) for mature females.*

And the equivalent raw data plots are:

***Figure 17.*** *Violin plot of *position* (y axis) function of *duration* (x axis) and *swelling* (panels) for mature females.*

The distribution of the random effects is:

***Figure 18.*** *Random effects: left = by individual, right = by year.*

#### Summaries per combinations of predictors:

Summaries of types of posture during feeding by age class.


| Sweling | # individuals | # Observations | # ‘up’ postures | % ‘up’ postures | # ‘down’ postures | % ‘down’ postures |
| --- | --- | --- | --- | --- | --- | --- |
| 1 | 7 | 381 | 189 | 49.6 | 192 | 50.4 |
| 2 | 5 | 158 | 109 | 69.0 | 49 | 31.0 |
| 3 | 6 | 264 | 175 | 66.3 | 89 | 33.7 |

Summaries of duration (mean, median, standard deviation and interquartile range) by type of posture.

| Posture | # individuals | # Observations | Mean | Median | SD | IQR |
| --- | --- | --- | --- | --- | --- | --- |
| down | 7 | 330 | 2.4 | 2 | 1 | 1.5 |
| up | 7 | 473 | 1.8 | 2 | 1 | 1.3 |

Summaries of duration (mean, median, standard deviation and interquartile range) by type of posture and age class.

| Posture | Swelling | # individuals | # Observations | Mean | Median | SD | IQR |
| --- | --- | --- | --- | --- | --- | --- | --- |
| down | 1 | 7 | 192 | 2.3 | 2 | 1.0 | 1.3 |
| down | 2 | 5 | 49 | 2.4 | 2 | 0.9 | 1.2 |
| down | 3 | 6 | 89 | 2.7 | 3 | 1.1 | 1.5 |
| up | 1 | 7 | 189 | 1.8 | 2 | 0.9 | 1.2 |
| up | 2 | 4 | 109 | 1.7 | 2 | 1.1 | 1.3 |
| up | 3 | 6 | 175 | 1.9 | 2 | 1.0 | 1.5 |

### Mature males only

#### Outliers:

We identified and removed 3 outliers; in order of removal, these are:

|  | date\_collection | subject | posture | duration | age | swelling | year\_collection |
| --- | --- | --- | --- | --- | --- | --- | --- |
| 738 | 2014-06-26 | 4 | up | 2.197225 | 18 | NA | 2014 |
| 705 | 2014-06-22 | 4 | up | 1.791759 | 18 | NA | 2014 |
| 2323 | 2018-06-17 | 4 | up | 1.609438 | 22 | NA | 2018 |

#### Summaries:

The distribution of postures across individuals (with column and row sums) is:

|  | down | up | Sum |
| --- | --- | --- | --- |
| **1** | 7 | 1 | 8 |
| **3** | 124 | 13 | 137 |
| **4** | 199 | 4 | 203 |
| **5** | 71 | 6 | 77 |
| **8** | 139 | 10 | 149 |
| **12** | 25 | 1 | 26 |
| **Sum** | 565 | 35 | 600 |

#### Retained *IV*s:

The potential IVs (each tested individually for their contribution to *posture*):

Individual contribution of all potential IVs to *posture*. We only keep the ones with a significant individual contribution (column *kept*) for further modelling.

| IV | beta | p | AIC | kept |
| --- | --- | --- | --- | --- |
| 1 | NA | NA | 287.2 | yes |
| age | 0.022 | 0.6911 | 289.1 | no |
| hierarchy | 0.116 | 0.4874 | 288.7 | no |
| duration | -1.344 | 0.0000 | 241.2 | yes |

#### Best model:

Please note that *year\_collection* as random effect has 0 variance and it can be eliminated from the model (thus, there is no `(1 | year_collection)` random effect in the formula).

The best model (with diagnostic plots) is:

posture ~ 1 + duration + (1 | subject)

|  | posture | | |
| --- | --- | --- | --- |
| Predictors | Log-Odds | CI | p |
| (Intercept) | -0.16 | -1.19 – 0.88 | 0.767 |
| duration | -1.52 | -2.00 – -1.04 | **<0.001** |
| Random Effects | | | |
| σ2 | 3.29 | | || τ00 subject | 0.69 | | || ICC | 0.17 | | || N subject | 6 | | || Observations | 600 | | |
| Marginal R2 / Conditional R2 | 0.406 / 0.509 | | |
| Deviance | 210.998 | | |
| AIC | 216.998 | | |
| log-Likelihood | -105.499 | | |

***Figure 19.*** *Model diagnostic plot (NB: this is on the *link scale*).*

***Figure 20.*** *Autocorrelation of the model’s residuals.*

The best model’s predictions are:

***Figure 21.*** *Predicted probability of *up* (y axis) function of *duration* (x axis) for mature males.*

And the equivalent raw data plots are:

***Figure 22.*** *Violin plot of *position* (y axis) function of *duration* (x axis) for mature males.*

The distribution of the random effects is:

***Figure 23.*** *Random effects: by individual.*

### Immatures only

#### Summaries:

The distribution of postures across individuals (with column and row sums) is:

|  | down | up | Sum |
| --- | --- | --- | --- |
| **5** | 58 | 9 | 67 |
| **6** | 102 | 43 | 145 |
| **9** | 49 | 4 | 53 |
| **11** | 69 | 14 | 83 |
| **12** | 56 | 13 | 69 |
| **14** | 3 | 0 | 3 |
| **15** | 77 | 78 | 155 |
| **16** | 132 | 115 | 247 |
| **17** | 31 | 26 | 57 |
| **21** | 56 | 62 | 118 |
| **Sum** | 633 | 364 | 997 |

#### Retained *IV*s:

The potential IVs (each tested individually for their contribution to *posture*):

Individual contribution of all potential IVs to *posture*. We only keep the ones with a significant individual contribution (column *kept*) for further modelling.

| IV | beta | p | AIC | kept |
| --- | --- | --- | --- | --- |
| 1 | NA | NA | 1216.3 | yes |
| age | 0.149 | 0.1127 | 1215.7 | no |
| sex | -0.310 | 0.6382 | 1218.0 | no |
| duration | -0.783 | 0.0000 | 1125.2 | yes |

#### Best model:

The best model (with diagnostic plots) is:

posture ~ 1 + duration + (1 | subject) + (1 | year\_collection)

|  | posture | | |
| --- | --- | --- | --- |
| Predictors | Log-Odds | CI | p |
| (Intercept) | 0.19 | -0.91 – 1.29 | 0.734 |
| duration | -0.78 | -0.95 – -0.61 | **<0.001** |
| Random Effects | | | |
| σ2 | 3.29 | | || τ00 subject | 0.98 | | || τ00 year\_collection | 0.50 | | || ICC | 0.31 | | || N subject | 10 | | || N year\_collection | 3 | | || Observations | 997 | | |
| Marginal R2 / Conditional R2 | 0.106 / 0.383 | | |
| Deviance | 1117.193 | | |
| AIC | 1125.193 | | |
| log-Likelihood | -558.596 | | |

***Figure 24.*** *Model diagnostic plot (NB: this is on the *link scale*).*

***Figure 25.*** *Autocorrelation of the model’s residuals.*

The best model’s predictions are:

***Figure 26.*** *Predicted probability of *up* (y axis) function of *duration* (x axis) for immatures.*

And the equivalent raw data plots are:

***Figure 27.*** *Violin plot of *position* (y axis) function of *duration* (x axis) for immatures.*

The distribution of the random effects is:

***Figure 28.*** *Random effects: left = by individual, right = by year.*

### All individuals

Please note that *swelling* and *hierarchy* are not defined for all types of individuals.

##### Outliers:

We identified and removed 5 outliers; in order of removal, these are:

|  | date\_collection | subject | posture | duration | age | age\_class | sex | year\_collection |
| --- | --- | --- | --- | --- | --- | --- | --- | --- |
| 738 | 2014-06-26 | 4 | up | 2.197225 | 18 | mature | M | 2014 |
| 705 | 2014-06-22 | 4 | up | 1.791759 | 18 | mature | M | 2014 |
| 1068 | 2014-07-15 | 5 | up | 2.564949 | 10 | mature | M | 2014 |
| 2323 | 2018-06-17 | 4 | up | 1.609438 | 22 | mature | M | 2018 |
| 135 | 2012-07-03 | 3 | up | 2.397895 | 11 | mature | M | 2012 |

##### Summaries:

The distribution of postures across individuals (with column and row sums) is:

|  | down | up | Sum |
| --- | --- | --- | --- |
| **1** | 7 | 1 | 8 |
| **2** | 17 | 44 | 61 |
| **3** | 124 | 12 | 136 |
| **4** | 199 | 4 | 203 |
| **5** | 129 | 14 | 143 |
| **6** | 102 | 43 | 145 |
| **7** | 107 | 239 | 346 |
| **8** | 139 | 10 | 149 |
| **9** | 49 | 4 | 53 |
| **10** | 75 | 64 | 139 |
| **11** | 69 | 14 | 83 |
| **12** | 81 | 14 | 95 |
| **13** | 60 | 50 | 110 |
| **14** | 3 | 0 | 3 |
| **15** | 77 | 78 | 155 |
| **16** | 132 | 115 | 247 |
| **17** | 31 | 26 | 57 |
| **18** | 8 | 6 | 14 |
| **19** | 38 | 22 | 60 |
| **20** | 25 | 48 | 73 |
| **21** | 56 | 62 | 118 |
| **Sum** | 1528 | 870 | 2398 |

##### Retained *IV*s:

The potential IVs (each tested individually for their contribution to *posture*):

Individual contribution of all potential IVs to *posture*. We only keep the ones with a significant individual contribution (column *kept*) for further modelling.

| IV | beta | p | AIC | kept |
| --- | --- | --- | --- | --- |
| 1 | NA | NA | 2579.3 | yes |
| sex | -1.679 | 0.0005 | 2569.2 | yes |
| age\_class | -0.686 | 0.0505 | 2577.5 | yes |
| age | 0.022 | 0.3328 | 2580.4 | no |
| duration | -0.718 | 0.0000 | 2403.2 | yes |

##### Best model:

The best model (with diagnostic plots) is:

posture ~ sex + duration + age\_class + (1 | subject) + (1 | year\_collection) + sex:duration + sex:age\_class

|  | posture | | |
| --- | --- | --- | --- |
| Predictors | Log-Odds | CI | p |
| (Intercept) | 0.13 | -0.77 – 1.02 | 0.7825 |
| sex [M] | 0.63 | -0.51 – 1.76 | 0.2789 |
| duration | -0.56 | -0.68 – -0.43 | **<0.001** |
| age\_class [mature] | 1.03 | 0.08 – 1.98 | **0.0337** |
| sex [M] \* duration | -0.72 | -1.01 – -0.42 | **<0.001** |
| sex [M] \* age\_class [mature] | -2.31 | -3.60 – -1.02 | **0.0004** |
| Random Effects | | | |
| σ2 | 3.29 | | || τ00 subject | 0.61 | | || τ00 year\_collection | 0.16 | | || ICC | 0.19 | | || N subject | 21 | | || N year\_collection | 3 | | || Observations | 2398 | | |
| Marginal R2 / Conditional R2 | 0.402 / 0.516 | | |
| Deviance | 2309.922 | | |
| AIC | 2325.922 | | |
| log-Likelihood | -1154.961 | | |

The *p*-values of the interactions are given below:

Significance of interaction terms.

| Interaction | *p*-value |
| --- | --- |
| sex:age\_class | 0.0016 |
| sex:duration | 0.0000 |

***Figure 29.*** *Model diagnostic plot (NB: this is on the *link scale*).*

***Figure 30.*** *Autocorrelation of the model’s residuals.*

The best model’s predictions are:

***Figure 31.*** *Predicted probability of *up* (y axis) function of *duration* (x axis), *age\_class* (panels) and *sex* (colors) for all individuals in all years.*

And the equivalent raw data plots are:

***Figure 32.*** *Violin plot of *position* (y axis) function of *duration* (x axis), *age\_class* and *sex* (panels) for all individuals in all years.*

The distribution of the random effects is:

***Figure 33.*** *Random effects: left = by individual, right = by year.*

#### Summaries per combinations of predictors:

Summaries of types of posture during feeding by age class.


| Age class | # individuals | # Observations | # ‘up’ postures | % ‘up’ postures | # ‘down’ postures | % ‘down’ postures |
| --- | --- | --- | --- | --- | --- | --- |
| immature | 10 | 997 | 364 | 36.5 | 633 | 63.5 |
| mature | 13 | 1406 | 511 | 36.3 | 895 | 63.7 |

Summaries of types of posture during feeding by sex.


| Sex | # individuals | # Observations | # ‘up’ postures | % ‘up’ postures | # ‘down’ postures | % ‘down’ postures |
| --- | --- | --- | --- | --- | --- | --- |
| F | 12 | 1423 | 723 | 50.8 | 700 | 49.2 |
| M | 9 | 980 | 152 | 15.5 | 828 | 84.5 |

Summaries of types of posture during feeding by age class and sex.


| Age class | Sex | # individuals | # Observations | # ‘up’ postures | % ‘up’ postures | # ‘down’ postures | % ‘down’ postures |
| --- | --- | --- | --- | --- | --- | --- | --- |
| immature | F | 5 | 620 | 250 | 40.3 | 370 | 59.7 |
| immature | M | 5 | 377 | 114 | 30.2 | 263 | 69.8 |
| mature | F | 7 | 803 | 473 | 58.9 | 330 | 41.1 |
| mature | M | 6 | 603 | 38 | 6.3 | 565 | 93.7 |

Summaries of duration (mean, median, standard deviation and interquartile range) by type of posture.

| Posture | # individuals | # Observations | Mean | Median | SD | IQR |
| --- | --- | --- | --- | --- | --- | --- |
| down | 21 | 1528 | 2.3 | 2 | 1.0 | 1.3 |
| up | 20 | 875 | 1.7 | 2 | 0.9 | 1.2 |

Summaries of duration (mean, median, standard deviation and interquartile range) by type of posture and age class.

| Posture | Age class | # individuals | # Observations | Mean | Median | SD | IQR |
| --- | --- | --- | --- | --- | --- | --- | --- |
| down | immature | 10 | 633 | 2.2 | 2 | 1.0 | 1.2 |
| down | mature | 13 | 895 | 2.4 | 2 | 1.0 | 1.5 |
| up | immature | 9 | 364 | 1.6 | 2 | 0.8 | 1.0 |
| up | mature | 13 | 511 | 1.8 | 2 | 1.0 | 1.3 |

Summaries of duration (mean, median, standard deviation and interquartile range) by type of posture and sex.

| Posture | Sex | # individuals | # Observations | Mean | Median | SD | IQR |
| --- | --- | --- | --- | --- | --- | --- | --- |
| down | F | 12 | 700 | 2.2 | 2 | 1.0 | 1.3 |
| down | M | 9 | 828 | 2.3 | 2 | 1.1 | 1.4 |
| up | F | 12 | 723 | 1.8 | 2 | 0.9 | 1.3 |
| up | M | 8 | 152 | 1.4 | 1 | 0.8 | 1.1 |

Summaries of duration (mean, median, standard deviation and interquartile range) by type of posture, age class and sex.

| Posture | Age class | Sex | # individuals | # Observations | Mean | Median | SD | IQR |
| --- | --- | --- | --- | --- | --- | --- | --- | --- |
| down | immature | F | 5 | 370 | 2.1 | 2 | 0.9 | 1.3 |
| down | immature | M | 5 | 263 | 2.3 | 2 | 1.0 | 1.3 |
| down | mature | F | 7 | 330 | 2.4 | 2 | 1.0 | 1.5 |
| down | mature | M | 6 | 565 | 2.4 | 2 | 1.1 | 1.4 |
| up | immature | F | 5 | 250 | 1.7 | 2 | 0.8 | 1.1 |
| up | immature | M | 4 | 114 | 1.4 | 1 | 0.8 | 1.1 |
| up | mature | F | 7 | 473 | 1.8 | 2 | 1.0 | 1.3 |
| up | mature | M | 6 | 38 | 1.3 | 1 | 0.7 | 1.1 |

### Effect of duration per individual

We used two ways to check the direction and strength of *duration* on *position* for each individual:

1. we performed a mixed-effects model of *posture* on *duration* allowing also the slope of *duration* to vary by *subject*:

glmer(posture ~ 1 + duration + (1 + duration | subject) + (1 | year\_collection)

and we obtained the following slopes of *duration* per *subject*:

|  | posture | | |
| --- | --- | --- | --- |
| Predictors | Odds Ratios | CI | p |
| (Intercept) | 1.66 | 0.82 – 3.38 | 0.162 |
| duration | 0.43 | 0.35 – 0.53 | **<0.001** |
| Random Effects | | | |
| σ2 | 3.29 | | || τ00 subject | 1.26 | | || τ00 year\_collection | 0.14 | | || τ11 subject.duration | 0.12 | | || ρ01 subject | 0.02 | | || ICC | 0.39 | | || N subject | 21 | | || N year\_collection | 3 | | || Observations | 2403 | | |
| Marginal R2 / Conditional R2 | 0.122 / 0.463 | | |
| Deviance | 2379.788 | | |
| AIC | 2391.788 | | |
| log-Likelihood | -1189.894 | | |

Multi-level logistic regression of `posture` on `duration` allowing random slopes for `duration` across individuals. We show the actual slope (beta) per individual.

|  | duration |
| --- | --- |
| 1 | -0.94 |
| 2 | -0.98 |
| 3 | -1.23 |
| 4 | -1.01 |
| 5 | -1.08 |
| 6 | -0.62 |
| 7 | -0.45 |
| 8 | -1.13 |
| 9 | -0.98 |
| 10 | -0.39 |
| 11 | -1.11 |
| 12 | -1.15 |
| 13 | -0.88 |
| 14 | -0.91 |
| 15 | -0.95 |
| 16 | -0.80 |
| 17 | -0.39 |
| 18 | -0.65 |
| 19 | -0.64 |
| 20 | -0.67 |
| 21 | -0.52 |

***Figure 34.*** *Random effects: left = by individual (intercepts and slopes; please note that these are different from the table above as we did not add the overall intercept), right = by year (intercepts).*

2. we performed separate “flat” (i.e., non-multilevel) logistic regressions of *posture* on *duration* for each subject individually (and for individual years, if a particular individual appears in more than a year), and we obtained the following slopes (with uncorrected *p*-values, as most do not survive correction due to the small sample sizes and large number of such cases):

Separate logistic regressions of `posture` on `duration` independently for each individual (and observation year). We show the beta and uncorrected p-value for `duration`.

| individual | betaduration | std.err(betaduration) | pduration |
| --- | --- | --- | --- |
| 16 | -0.81 | 0.16 | 0.0000 |
| 3 | -1.61 | 0.42 | 0.0001 |
| 5 | -1.32 | 0.33 | 0.0001 |
| 7 | -0.48 | 0.12 | 0.0000 |
| 8 | -1.37 | 0.44 | 0.0018 |
| 10 | -0.26 | 0.17 | 0.1282 |
| 2 | -2.01 | 0.58 | 0.0006 |
| 4 | -0.91 | 0.46 | 0.0480 |
| 18 | -0.45 | 0.46 | 0.3307 |
| 13 | -1.06 | 0.24 | 0.0000 |
| 12 | -1.41 | 0.41 | 0.0005 |
| 15 | -1.05 | 0.21 | 0.0000 |
| 1 | -43.95 | 15656.04 | 0.9978 |
| 6 | -0.55 | 0.21 | 0.0102 |
| 19 | -0.50 | 0.33 | 0.1351 |
| 14 | 0.00 | 54315.71 | 1.0000 |
| 11 | -1.42 | 0.49 | 0.0038 |
| 17 | 0.42 | 0.44 | 0.3427 |
| 21 | -0.40 | 0.22 | 0.0732 |
| 20 | -0.68 | 0.33 | 0.0369 |
| 9 | -0.67 | 0.70 | 0.3369 |

***Figure 35.*** *Individual estimates of the slope (blue circles) and its standard error (red lines) for each individual. Please note that the plot is limited t the [-4,+2] range to avoid being swamped by the huge estimates and standard errors of the two individuals with very skewed data (1 and 14).*

Please note that the 2nd method is not very reliable, as for some individuals the number of observations is very low and it treates each individul as independent…

Nevertheless, it can be seen that with both methods the slopes per individual are almost all negative, i.e., that duration has a negative influence on the probability of an “up” position for most individuals.

## Plots for the paper

Here we generate high-quality plots for the main paper.

### Full figure

This contains both the actual data and the model predictions, saved as `./Figure-full.eps` (EPS format) and `./Figure-full.tiff` (300DPI TIFF format)…

***Figure 36.*** *Visual representation of the results. The top row (panels **A** and **C**) shows the raw data, while the bottom row (panels **B** and **D**) shows the predictions from the regression models; the left-hand side (panels **A** and **B**) shows the results for all the individuals across all the years, sexes and maturity classes, while the right-hand side (panels **C** and **D**) shows the results for the mature females only. The top panels (**A** and **C**) display, for each type of position (‘up’ or ‘down’) on the vertical axis, the distribution of the log(duration), on the horizontal axis, corresponding to a given subgroup of individuals: maturity class (‘immature’ vs ‘mature’) by sex (‘F’ vs ‘M’) for panel **A**, and swelling (levels ‘1’, ‘2’ and ‘3’) for panel **C**; please note that the actual datapoints are jittered on the vertical axis for improved visibility. The bottom panels (**B** and **D**) display the probability (as a pecent, %) of adopting the ‘up’ position on the vertical axis, given log(duration) on the horizontal axis, corresponding to a given subgroup of individuals: maturity class (‘immature’ vs ‘mature’) by sex (‘F’ vs ‘M’) for panel **B**, and swelling (levels ‘1’, ‘2’ and ‘3’) for panel **D**.*

### Model predictions only

This contains only the model predictions, saved as `./Figure.eps` (EPS format) and `./Figure.tiff` (600DPI TIFF format)…

***Figure 37.*** *Same as above.*

---

1. For *factors* we use either *sum contrasts* (`contr.sum`) where the comparison is made with the grand mean of the variable, or, for *ordered factors*, we use *polynomial contrasts* (`contr.poly`) which, for 3-level factors (as here), encode the *linear* effect (defined as level 3 - level 1 and denoted by the suffix “.L”) and the quadratic effect (defined as level 1 + level 3 - level 2 and denoted by the suffix “.Q”). So, for the sum contrast a significant *p*-value means that the level is different from the grand mean, while for the plynomial result a significant effect will point to linear effect (.L) and/or to a significant deviation from linearity (positive means concave, negative means convex).↩︎
